# Burden of Infectious Diseases in Afghanistan in 1999 and 2023: Findings From the Global Burden of Disease 2023 Study

**DOI:** 10.7759/cureus.103884

**Published:** 2026-02-18

**Authors:** Ahmad Khan, Melanie Tidman

**Affiliations:** 1 Health Sciences, A.T. Still University, Albuquerque, USA

**Keywords:** afghanistan, dalys, infectious diseases, lower-income countries, ylds, ylls

## Abstract

Afghanistan faces a critical health challenge due to different diseases, including diseases caused by infectious diseases, worsened by the structural limitations typical pattern in low- and middle-income countries. This secondary analysis of modeled 2023 estimated assessed data to evaluate changes in Disability-Adjusted Life Years (DALYs), Years Lived With Disability (YLDs), and Years of Life Lost (YLLs) in Afghanistan in 1999 and 2023. We used multiple regression using SPSS Software Version 28 (IBM Corp., Armonk, NY), and the results revealed that the Afghan population has experienced a statistically significant improvement in health outcomes for diseases caused by infectious diseases, with reductions in DALYs (64.8%), YLDs (47.9%), and YLLs (65.0%). The results were consistent with those of neighboring countries, suggesting the effectiveness of access to health sector services. The decrease in the burden of infectious diseases in Afghanistan can be attributed to improved access to curative medicine services and proactive public health measures, such as vaccination programs, access to clean sanitation systems, and better health literacy. The result did not show statistically significant gender differences in YLLs, YLDs, or DALYs, which challenges the conventional understanding of gender-based health disparities in access to care. The sustainability of Afghanistan's health gains can be threatened if persistent political instability and critical gaps in medical infrastructure continue.

## Introduction

Infectious diseases have long been a critical public health concern in lower-income countries such as Afghanistan. Afghanistan has grappled with a fragile healthcare system for the last couple of decades. Since the early 1990s, it has experienced persistent socio-political instability and humanitarian crises, which have contributed to the disruption of the delivery of healthcare services and a high burden of communicable diseases [1].

The Institute for Health Metrics and Evaluation (IHME) Global Burden of Disease (GBD) 2023 Study is a well-known source that provides information on epidemiological patterns of infectious diseases and their impacts on the global population by country [2]. For this secondary analysis, the focus on the Afghan population will include a comprehensive assessment of morbidity and mortality rates attributable to infectious diseases [2].

Various pathogens, including bacteria, viruses, and parasites, can cause infectious diseases, which account for a substantial share of healthcare costs in lower-income countries such as Afghanistan [3]. Conditions such as tuberculosis, malaria, HIV/AIDS, and other prevalent infectious diseases lead to considerable health challenges, exacerbated by various determinants including inadequate nutritious food, poor sanitation, poor water quality, and lack of access to healthcare services and preventive care measures [4]. The connection of these health determinants with infectious diseases surpasses the susceptibility of the Afghan population to infectious diseases and increased mortality.

Afghanistan has instituted numerous public health interventions to combat infectious diseases, yet their effectiveness has varied from 1999 to 2023 [5]. At the same time, the local government and international nongovernment organizations have increased their efforts to improve vaccination coverage and other infectious disease prevention programs [6]. Yet, persistent barriers, including cultural resistance, political instability, and disruptions in healthcare delivery caused by political and social conflict, have hindered sustained improvements in healthcare services in the country [7].

Within the GBD 2023 framework, communicable diseases are categorized as Level 1 causes. Under the etiology section, available infectious diseases on the list were selected. Infectious diseases entail a range of specific conditions, including but not limited to HIV/AIDS, tuberculosis, malaria, hepatitis, and respiratory infections, and their corresponding International Classification of Diseases (ICD) codes are documented in the GBD database [8]. The burden of infectious diseases is quantified using Disability-Adjusted Life Years (DALYs), which are computed as the sum of Years of Life Lost (YLLs) and Years Lived With Disability (YLDs) [9]. This calculation has both premature mortality and non-fatal health outcomes attributable to communicable diseases. The GBD 2023 provides comprehensive estimates of incidence, prevalence, mortality, and disability due to various diseases, standardized by location, age, and sex across 204 countries and regions, including Afghanistan (https://vizhub.healthdata.org) [10].

The data from the GBD 2023 Study highlight epidemiological shifts in various communicable diseases in Afghanistan and provide crucial insights into the dynamics of the overall burden and the underlying contributors that shape these shifts in infectious disease trends [10]. This secondary analysis of the GBD 2023 estimates aims to examine the findings from the GBD 2023 Study regarding the burden of infectious diseases contrast in Afghanistan in 1999 and in 2023. By examining the burden of infectious diseases over this period, a comprehensive understanding of the current state of communicable diseases in Afghanistan can be achieved.

## Materials and methods

The objective of this secondary data analysis is to assess the burden of infectious diseases in Afghanistan in 1999 and 2023, and across genders. This secondary data analysis investigates the burden of infectious diseases in Afghanistan for 1999 and 2023, using GBD 2023 data, which provide comprehensive health metrics, including incidence, prevalence, mortality, YLLs, YLDs, and DALYs related to infectious pathogens [11]. The GBD 2023 data encompass a wide range of diseases and span demographics across multiple countries and regions. The data for the GBD 2023 are derived from systematic reviews, population surveys, vital registration data, and verbal autopsy records (VARs) [12].

Data quality control and data collection

In the current study, the GBD 2023 database is used to analyze rates reflecting the burden of infectious pathogens in Afghanistan in 1999 and 2023. The GBD 2023 uses a well-structured quality control process that begins by assessing data availability and sources, including vital registration systems (VRSs), health surveys, and other health-related databases, to ensure high-quality data [12]. These sources undergo a validation process where metrics such as completeness, consistency, and reliability are examined. Furthermore, the GBD 2023 framework uses multiple imputation techniques to address missing data and model uncertainties, enhancing the overall quality of health estimates [13].

Mortality from communicable diseases is categorized according to the International Statistical Classification of Diseases (ICD) and Related Health Problems [14]. The assessment of the underlying causes of communicable diseases was conducted using automated text-mining algorithms applied to death certificates and VARs. The Cause of Death Ensemble model (CODEm) was used to synthesize available data and provide a meta-analytical approach, selecting the most reliable models that best fit the diverse data pool [15]. The burden of communicable diseases was estimated using the Disease Model for Meta-Regression (DisMod-MR 2.1) modeling software (IHME, Seattle, WA). This Bayesian geospatial modeling tool uses epidemiological data about disease parameters, demographic variables, and geospatial contexts to estimate disease prevalence and incidence rates [16].

DALYs are used in this analysis as a composite measure to capture both mortality and morbidity related to communicable diseases. Components of DALYs are YLLs and YLDs. YLLs represent the years lost due to premature mortality [17]. YLL is calculated as the product of the estimated number of deaths due to a specific disease and the standard life expectancy at the age of death. YLL allows quantification of the impact of early death on overall population health. YLDs account for the years individuals live with a disability stemming from a disease [18]. YLDs are calculated by multiplying the prevalence of specific disease outcomes by their corresponding disability weights, which are standardized values ranging from zero (full health) to one (death), which reflect the severity of the health consequences individuals with diseases experience [19]. GBD uses different age groups and age-standardized rates to assess differences in age distributions across populations in Afghanistan, reported per 100,000 individuals. The age-standardized rates indicate the overall burden of communicable diseases, adjusted for age, ensuring any differences in population are not a result of variation in the age composition [20,21].

## Results

Multiple regression was used to assess the relationships between the predictors (gender and year) and the dependent variables DALYs, YLDs, and YLLs, using SPSS Software Version 28 (IBM Corp., Armonk, NY). Multiple regression analysis helped quantify the effects of each predictor while controlling for the others, providing insight into patterns and trends in the burden of infectious diseases over time. Gender and year were used as predictors to assess changes over time.

The multiple regression analysis indicates a negative trend over time related to DALYs (p < 0.001), YLDs (p = 0.021), and YLLs (p < 0.001). The significant negative coefficients for 1999 and 2023 in Afghanistan across all three models (DALYs, YLDs, and YLLs) suggest that from 1999 to 2023, there was a statistically significant reduction in the burden of infectious pathogens. DALYs with a decrease of approximately 190.293, YLDs with a decline of 1.124, and YLLs with a decrease of 7.882, leading to an improvement in overall health status, fewer years lived with disabilities, and a reduction in premature mortality of Afghans associated with infectious pathogens (Table 1).

**Table 1:** Summary of Regression Analysis Coefficient for DALYs, YLDs, and YLLs This table presents the results of regression analyses assessing the relationships among various predictors (gender and year) and the dependent variables DALYs, YLDs, and YLLs, for infectious pathogens. The table has unstandardized coefficients (B), standard errors (Std. Error), standardized coefficients (Beta), t-values (t), and associated p-values. The analyses revealed a statistically significant reduction in the burden of infectious pathogens. However, gender does not show a significant effect on any of the dependent variables DALYs, YLDs, or YLLs, as evidenced by p-values greater than 0.05 in all instances. a) Dependent variable: DALYs value. b) Dependent variable: YLDs value. c) Dependent variable: YLLs value. DALYs, Disability-Adjusted Life Years; YLDs, Years Lived With Disability; YLLs, Years of Life Lost

Coefficients						
Model		Unstandardized Coefficients		Standardized Coefficients	t	p-Value
		B	Std. Error	Beta		
a	(Constant)	494.163	104.606	-	4.724	<0.001
	Year	-190.293	52.303	-0.299	-3.638	<0.001
	Gender	-3.666	32.029	-0.009	-0.114	0.909
b	(Constant)	3.434	0.961	-	3.573	<0.001
	Year	-1.124	0.48	-0.197	-2.34	0.021
	Gender	0.051	0.294	0.014	0.172	0.864
c	(Constant)	16057.738	4361.596	-	3.682	<0.001
	Year	-7.882	2.169	-0.299	-3.635	<0.001
	Gender	-3.716	31.872	-0.01	-0.117	0.907

DALYs, YLDs, and YLLs coefficients indicate progress in reducing both morbidity and mortality related to infectious diseases in Afghanistan, and their negative trends for 1999 to 2023, which are statistically significant with a p-value of less than 0.05, meaning we can be reasonably confident that these trends are not due to random fluctuations but rather indicate an improvement in health outcomes. However, gender does not show a significant effect on any of the dependent variables, implying that gender differences have little to no influence on the outcomes measured in this analysis.

**Figure 1:**
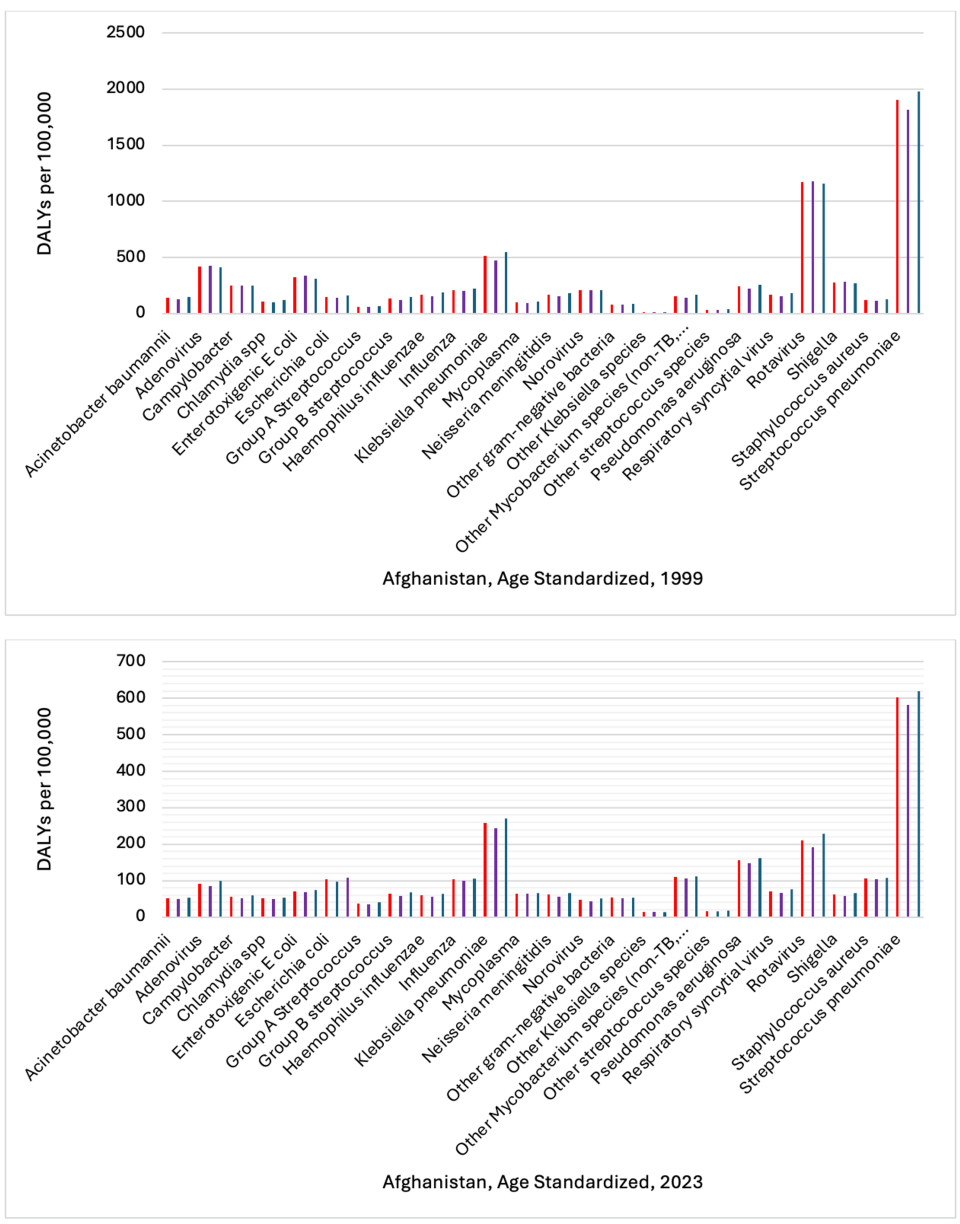
Comparison of Disability-Adjusted Life Years (DALYs) by Gender: 2023 vs. 1999 This figure shows age-standardized DALYs rate per 100,000 population for 24 infectious diseases in Afghanistan in 1999 and 2023. The vertical axis represents the rate of DALYs. The bars are categorized by sex, with color coding as follows: red denotes both sexes combined, purple denotes females, and dark blue denotes males. Each bar corresponds to a specific infectious disease. Reference: [22]

**Figure 2:**
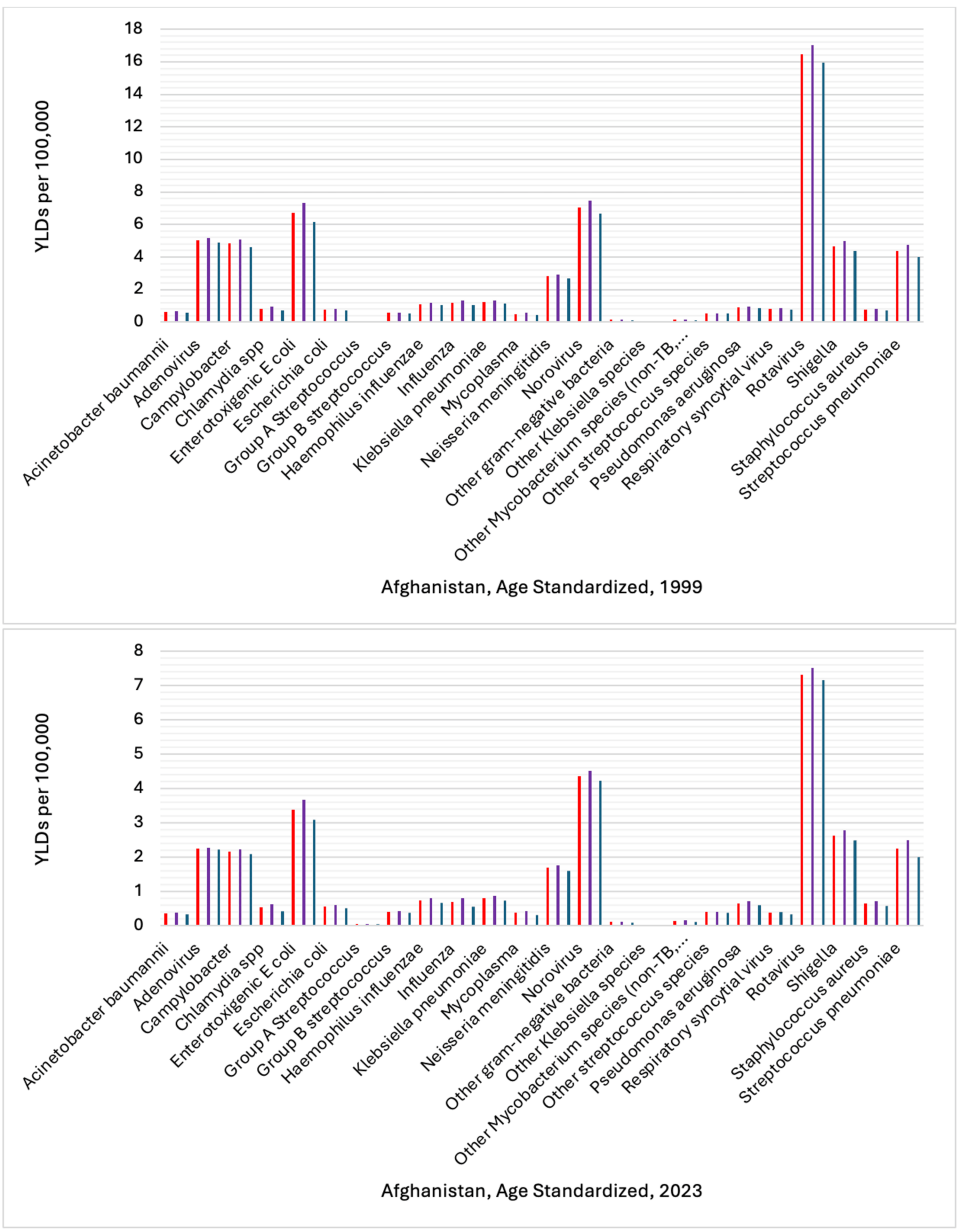
Comparison of Years Lived With Disability (YLDs) by Gender: 2023 vs. 1999 This figure shows age-standardized YLD rates per 100,000 population for 24 infectious diseases in Afghanistan, 1999 and 2023. The vertical axis represents the rate of YLDs. The bars are categorized by sex, with color coding as follows: red denotes both sexes combined, purple denotes females, and dark blue denotes males. Each bar corresponds to a specific infectious disease. Reference: [22]

**Figure 3:**
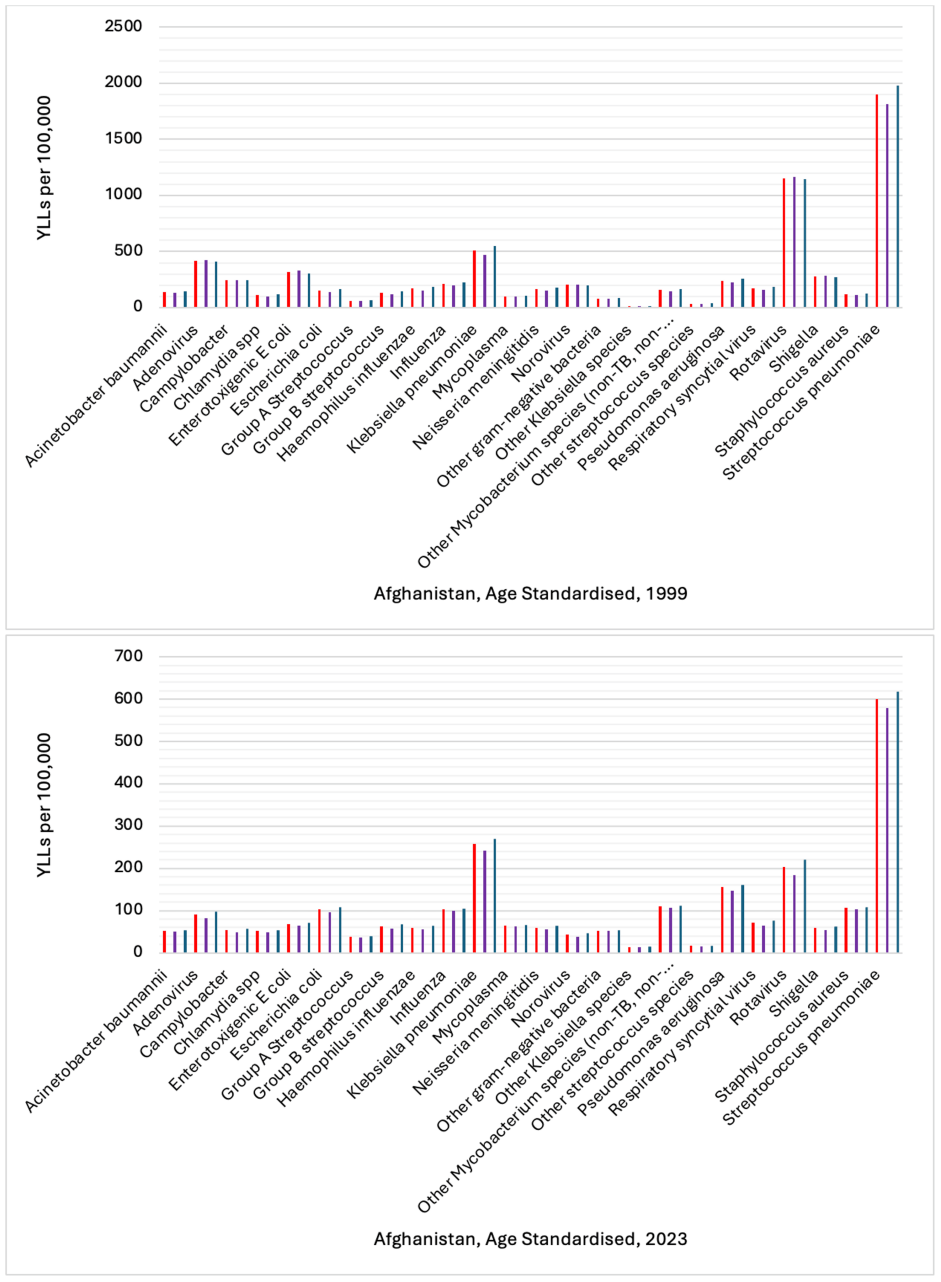
Comparison of Years of Life Lost (YLLs) by Gender: 2023 vs. 1999 This figure shows age-standardized YLL rates per 100,000 population for 24 infectious diseases in Afghanistan in 1999 and 2023. The vertical axis represents the rate of YLLs. The bars are categorized by sex, with color coding as follows: red denotes both sexes combined, purple denotes females, and dark blue denotes males. Each bar corresponds to a specific infectious disease. Reference: [22]

In setting up the analysis of health data from the IHME for 1999 and 2023, in the display section, etiology was selected, and in the cause section, communicable, maternal, neonatal, and nutritional diseases were selected for the location Afghanistan. The selected key measures were DALYs, YLDs, and YLLs. In the age section, standardized age was selected to ensure findings are comparable across age groups, and gender (males, females), and both sexes were selected. Additionally, the rate per 100,000 population was selected to provide a clear understanding of the burden of infectious pathogens in Afghanistan in 1999 and 2023 (Figures 1-3).

The sum of DALY rates per 100,000 for females, males, and both sexes for 24 infectious diseases was 21,434.20 in 1999, and in 2023, it decreased to 7,545.32, representing a reduction of approximately 64.8%. In 1999, the sum of YLD rates per 100,000 was 186.47, and by 2023, it had dropped to 98.90, resulting in a reduction of about 47.9%. The sum of YLLs rate per 100,000 in 1999 was 21,247.73. In 2023, this was reduced to 7,446.42, a decrease of approximately 65.0%.

## Discussion

In low- and middle-income countries, such as Afghanistan, the burden of infectious diseases presents an ongoing, significant public health threat. This review of the GBD 2023 database analyzed trends in DALYs, YLDs, and YLLs attributed to infectious diseases in 1999 and 2023, using GBD study data. The multiple regression analysis revealed decreases in YLLs, YLDs, and DALYs, suggesting better infectious disease outcomes for the Afghan population over 24 years.

The downward trend, specifically, the reductions in DALYs (64.8%), YLDs (47.9%), and YLLs (65.0%), indicates that, despite ongoing challenges in the healthcare system, public health interventions and improvements appear to be producing positive results. The reduction observed in Afghanistan is consistent with results from similar studies conducted in neighboring countries with lower income levels. For example, recent studies from Pakistan show that improved vaccination coverage and enhanced infectious disease management and prevention have reduced morbidity and mortality from infectious diseases [23].

The decrease in mortality and morbidity rates observed in the analysis of the GBD 2023 data for Afghanistan may be due to multiple health initiatives aimed at controlling the spread of infectious diseases [24]. Examples include effective and accessible health measures such as widespread vaccination campaigns, improved sanitation, initiatives to address health literacy, and on-time healthcare services accessibility, which have been essential in reducing the infectious disease burden in many countries, including Afghanistan [25]. A study conducted in Bangladesh indicated that community health interventions are effective, substantially improving public health outcomes, and that these improvements require implementing enhancements such as more effective disease surveillance and updated management protocols [26]. The findings indicated positive outcomes similar to those observed in Afghanistan.

However, no significant association was found between gender and YLLs, YLDs, or DALYs of infectious diseases in Afghanistan. This finding is notable because it contrasts with a portion of the existing research that points to gender-based health disparities, particularly in healthcare access and engagement with the healthcare system [27-29]. For example, studies from India have indicated that women have challenges in accessing timely healthcare that could exacerbate the disease burden of infection and other diseases [30,31]. The lack of a statistically significant gender difference reflected in the GBD 2023 data may indicate that the burden of infectious diseases was the same for both men and women in Afghanistan.

Limited infrastructure and instability can threaten the development of key public health programs. A study by Hibbert et al. assessing health systems in lower-income countries similar to Afghanistan indicated that maintaining and enhancing healthcare infrastructure, personnel, and systems is crucial to safeguard the progress achieved [32,33]. Additionally, continuous assessment of health impacts is crucial to ensure that progress is consistently supported over the long term.

Limitations and strengths

Using DALYs, YLLs, and YLDs for 1999 and 2023 provides valuable information. However, the GBD study has some limitations, particularly in analyzing trends over an extended period across different demographics in Afghanistan. The GBD data are gathered from various data sources, each with varying levels of reliability. Missing or underestimated data in certain parts of Afghanistan can skew results. Changes in data collection methods or definitions over time (1999 and 2023) can affect the comparability of data. The finding that no statistically significant difference was detected across genders may oversimplify the complex ways in which infectious diseases affect men and women, which require further investigation. Conversely, GBD 2023 data on DALYs, YLLs, and YLDs provide rates reflecting the burden of infectious diseases in Afghanistan for the period 1999-2023, offering a longitudinal perspective on trends and highlighting how these rates have shifted. The results indicate potential areas for further research, such as local demographics and disease-specific trends, which will facilitate future studies.

## Conclusions

The analysis of GBD 2023 data indicated that between 1999 and 2023, people in Afghanistan experienced substantial improvements in reducing the burden of infectious diseases, as evidenced by lower YLLs, YLDs, and overall DALYs. Access to health care services and public health initiatives, such as immunization and sanitation measures, has improved, leading to favorable health outcomes, even amid ongoing political and economic constraints and limited healthcare infrastructure. The apparent parity in infectious diseases’ health outcomes between Afghan females and males warrants further investigation into the root causes in the future. Afghanistan needs sustained funding for healthcare infrastructure and community programs to secure and build upon recent health gains, ensuring long-term benefits for the Afghan population.
